# Mapping the use of large language models in hiring decisions: a scoping review

**DOI:** 10.3389/frai.2026.1798519

**Published:** 2026-03-13

**Authors:** Arpit Tripathi, Ankit Tripathi, Frantisek Darena, Pawan Kumar Mishra

**Affiliations:** Faculty of Business and Economics, Mendel University in Brno, Brno, Czechia

**Keywords:** algorithmic decision-making, generative AI, hiring, human resource management, large language models, recruitment, scoping review, socio-technical systems

## Abstract

Large language models (LLMs) are increasingly being explored and deployed across recruitment and selection processes, reshaping how hiring decisions are supported, communicated, and justified. Unlike earlier algorithmic hiring tools, LLMs operate through language-mediated interaction, influencing interpretive and evaluative layers of decision-making. This scoping review maps the academic literature on LLMs in hiring to examine (i) where and how these systems are applied across the hiring pipeline, (ii) what forms of evidence and outcomes are assessed, (iii) which risks and mitigation strategies are documented, and (iv) how disciplinary structures shape research focus. Following PRISMA-ScR guidelines, we synthesize research published between 2018 and 2026 across multiple disciplines using a transparent, lexicon-based coding approach. The results reveal a rapidly expanding but uneven literature, characterized by concentration in early hiring stages, selective outcome measurement favoring efficiency and performance, high awareness of ethical risks with limited empirical validation of controls, and structurally constrained interdisciplinarity. The review highlights key gaps and provides a foundation for future interdisciplinary and field-based research on responsible LLM use in hiring.

## 1 Introduction

Hiring decisions occupy a central place in organizational system. They shape individual careers and influence organizational effectiveness. They also contribute to the reproduction or disruption of social inequality in labor markets. Hiring is often treated as a rational and regulated organizational function. However, several studies report that recruitment and selection outcomes remain systematically stratified by gender, age, ethnicity, and socioeconomic background (Bertrand and Mullainathan, 2004; Neumark, 2018; Pager and Shepherd, 2008; Quillian et al., 2017). These disparities suggest that hiring is not only a technical exercise. It is also a socially embedded process in which power, interpretation, and institutionalized expectations matter.

Hiring can also be seen as a socio-technical decision system in which technologies, formal procedures, human judgment, and organizational norms interact and shape outcomes (Orlikowski and Iacono, 2001; Trist and Bamforth, 1951). The Decisions are rarely the product of neutral assessment alone. They emerge through ongoing processes of interpretation and sense making. These decisions are often shaped by both formal evaluation procedures and subjective judgment in personnel selection processes (Highhouse, 2008). Organizational actors evaluate information, construct narratives about candidates, and justify choices to themselves and others (Weick, 1995). This makes hiring especially sensitive to technologies that intervene in how candidate information is produced, organized, and understood.

The earlier generations of hiring technologies mainly consisted of rule-based applicant tracking systems (ATS) or narrowly trained predictive machine learning (ML) models. LLMs differ in a fundamental way. They operate through language. They are increasingly used to parse and summarize résumés, generate interview questions, simulate candidate interactions, draft evaluative narratives, and documentation and compliance-related work (Bender et al., 2021; Bommasani et al., 2022). LLMs intervene in discursive spaces where meaning, legitimacy, and accountability are constructed. Even when final hiring authority remains with human decision-makers, these systems can influence how candidates are described, compared, and discussed. From a sense making perspective, LLMs shape the frames through which evaluators interpret candidate information (Weick, 1995). It also raises new questions about authority, responsibility, and bias in hiring.

Research on LLMs in hiring has grown rapidly (though fragmented and uneven). The reported contributions are conceptual or exploratory, rely on simulations or synthetic data, focus mainly on system design rather than organizational use in practice (Dietvorst et al., 2015). Existing empirical studies employ heterogeneous outcome measures and disciplinary traditions. This limits comparability and makes cumulative synthesis difficult. Under these conditions, a conventional systematic review or meta-analysis is not appropriate. We therefore adopt a scoping review approach following PRISMA-ScR guidelines (Tricco et al., 2018). Our aim is to map the existing body of research on LLMs in hiring. Scoping reviews are well suited to research areas in which concepts are still evolving. They are appropriate when terminology is unsettled and empirical maturity varies (Arksey and O’Malley, 2005; Munn et al., 2018). These conditions apply to scholarship on LLM-based hiring. Since the widespread diffusion of generative AI systems after 2022, relevant work has appeared across computer science, information systems, management, psychology, social sciences, medicine, and law. This literature draws on different theoretical traditions and prioritize different outcomes. They employ diverse methodological approaches. Bringing this dispersed body of work into a coherent analytical map is both challenging and necessary.

We do not seek to estimate effect sizes or establish causal claims. Instead, we identify where along the hiring pipeline LLMs are deployed and for what purposes. We characterize the types of evidence and study designs used. We document reported risks and proposed mitigation strategies. We also examine the disciplinary composition and temporal development of the field.

This review addresses four research questions.

*RQ1*: In which hiring stages are LLMs used, and for what tasks?

*RQ2*: What outcomes are assessed and which study designs dominate literature?

*RQ3*: What risks are documented, and which mitigation strategies are proposed or empirically tested?

*RQ4*: How is the literature structured across disciplines and how does disciplinary orientation shape research focus?

The review contributes to literature in three ways. First, it offers a stage-wise socio-technical map of LLM applications across the hiring pipeline, highlighting the stage where language-mediated automation is concentrated (RQ1). Second, it provides a theory-informed typology of evidence and outcomes, revealing systematic misalignments between how hiring is understood theoretically and how LLMs are evaluated empirically (RQ2–RQ3). Third, it analyzes disciplinary structure and epistemic fragmentation, demonstrating how disciplinary boundaries shape research agendas and constrain integrative theory-building (RQ4).

## 2 Methods

### 2.1 Protocol and reporting standard

The review was conducted as per PRISMA-ScR reporting guidelines (Tricco et al., 2018). A structured protocol was developed prior to analysis specifying eligibility criteria, search strategy, screening procedures, and data-charting variables. Using the PCC (population, concept and context) framework, eligibility criteria were defined across three interconnected dimensions (Pollock et al., 2023):

- Population: Job applicants, candidates, recruiters, HR professionals, hiring managers, and organizations.
- Concept: Large language models or generative AI systems explicitly used for hiring-related decision support or automation.
- Context: Recruitment, selection, interviewing, and onboarding processes.

Studies were included if they examined, evaluated, or proposed LLM-based tools directly relevant to hiring decisions.

Search was conducted in Scopus, selected for its broad interdisciplinary coverage and consistent bibliographic metadata (Chadegani et al., 2013). The core search strategy combined terms related to LLM (e.g., large language model, generative AI, transformer) with hiring related terms (e.g. recruitment, selection, interview, resume screening) using appropriate discipline search syntax. This review included journal articles and review articles. The time window spanned from 2020 to 2025 reflecting the emergence of transformer-based architecture and their accelerated diffusion following 2022. Detailed search query, PRISMA ScR flowchart and inclusion exclusion table in Supplementary material.

### 2.2 Screening, charting, and coding

Records were de-duplicated across discipline-specific exports. Data were charted at the paper level, extracting bibliographic metadata (publication year, discipline, outlet), application characteristics (hiring stage, task type), outcomes assessed, risks discussed, and governance mechanisms proposed.

All analytical coding followed a lexicon-based, rule-driven approach:

- Coding relied exclusively on explicit terms appearing in titles, abstracts, or author keywords.
- Categories were coded using binary indicators (presence/absence) or proportional measures (percentage shares).
- No machine learning, topic modeling, or latent inference techniques were applied, in line with scoping review best practice and to maximize interpretability.

Complete lexicon tables used for coding outcomes, risks, and conceptual focus are provided in the Supplementary material.

Results were synthesized using: Descriptive mapping, Cross-tabulations and heat maps, Pre/post-2022 comparisons to capture shifts following the widespread diffusion of generative AI. No causal claims were made. Findings were interpreted as patterns of research attention and emphasis, rather than evidence of effectiveness, harm or organizational impact.

## 3 Results and discussion

**Figure 1:**
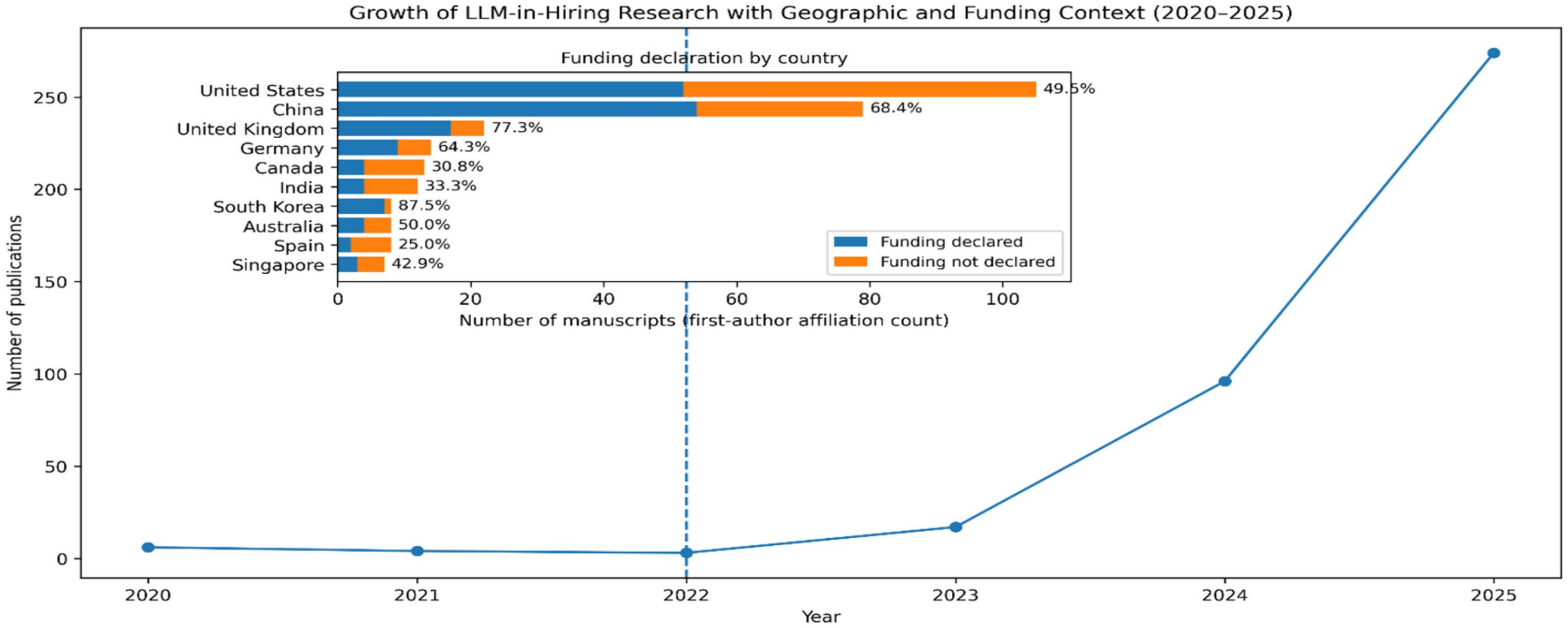
Growth of LLM in hiring research. Source Scopus data (2020–2025). Percentage indicate percentage of funded studies derived from dedication to project in the manuscript.

Scoping reviews describe the scope and characteristics of research rather than test causal effects (Arksey and O’Malley, 2005; Tricco et al., 2018). As presented in Figure 1, a sudden surge in number of publications can be clearly seen following the AI diffusion (post 2022). Moreover, looking at the funded studied vs. unfunded studies (based on the funding statement declared in the manuscript), the maximum number of funded studies were reported from south Korea, followed by U. K., China, and Germany (extremes represented by South-Korea and Spain).

**Figure 2:**
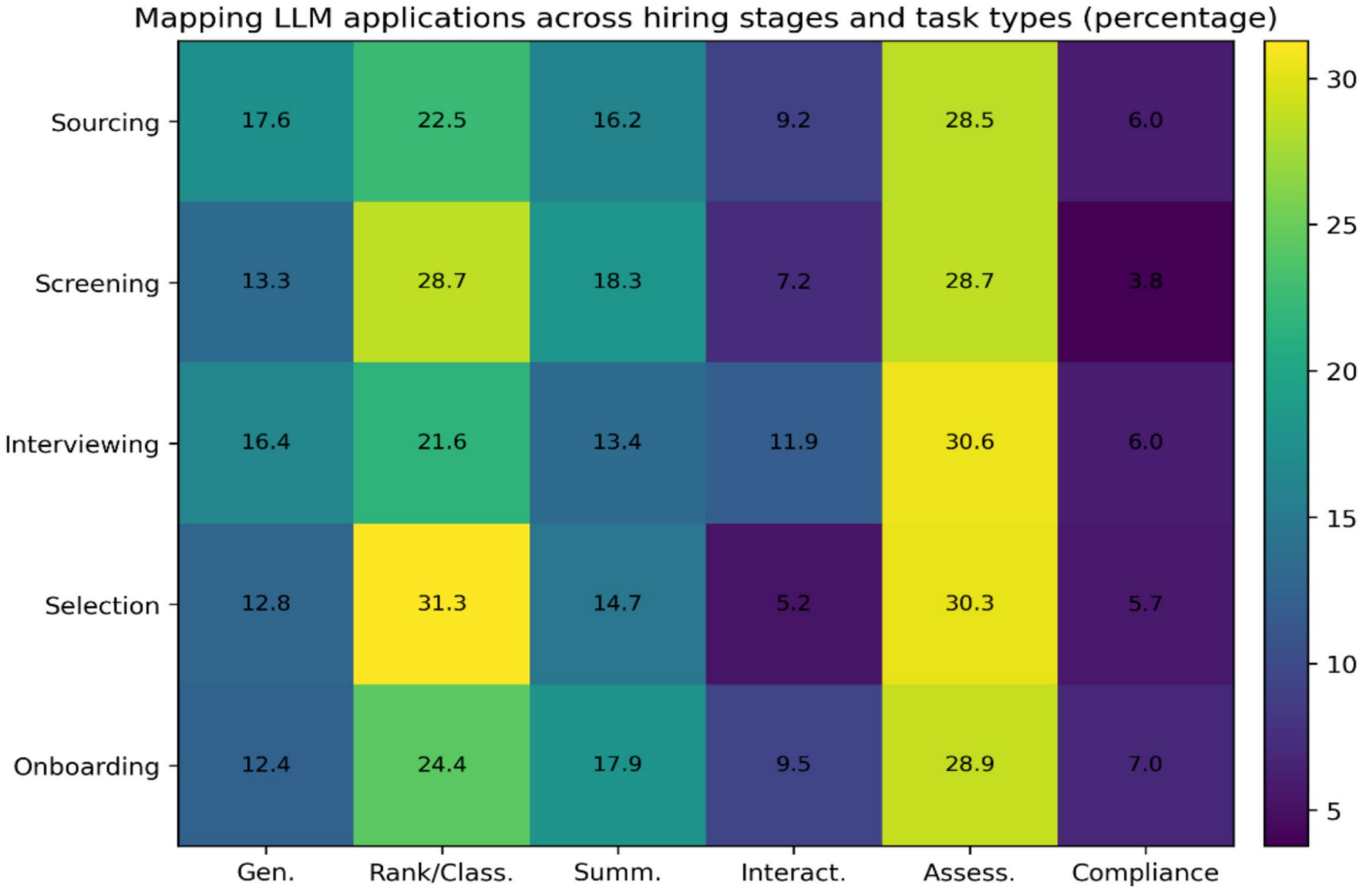
Hiring stage at which LLM is used (Gen., generative; Class., classification; Summ., summarization; Interact., Interaction; Assess., assessment). Lexicon coding table- Supplementary file.

This trend does not follow the quantitative trend of total number publications (Extremes represented by the USA and Singapore). This difference may reflect variation in national funding priorities, government support for AI research, and institutional incentives for funded projects (Waltman, 2016; Zupic and Čater, 2015). Figure 2 presents the mapping of LLM applications across the hiring pipeline. It reveals a clear concentration of research attention in screening and interviewing stages. This pattern reflects a long-standing organizational tendency to automate tasks perceived as repetitive, high-volume, and operationally costly (Autor et al., 2003).

From a socio-technical systems perspective, LLMs are primarily positioned within the technical subsystem of hiring. In contrast, the social subsystem, including judgment, accountability, and organizational responsibility, remains comparatively under examined (Baxter and Sommerville, 2011; Trist and Bamforth, 1951). This concentration of LLM use in screening and interviewing suggests that organizations are integrating LLMs primarily to support structured information processing rather than to replace human evaluative authority. This pattern reflects bounded rationality and risk-sensitive technology adoption, where automation is introduced first in tasks perceived as operationally reversible (March and Simon, 1958; Parasuraman and Riley, 1997). Future research should examine whether LLM deployment expands into higher-consequence decision stages such as final selection and onboarding, and how organizational governance structures shape the scope and autonomy of LLM-supported hiring decisions.

**Figure 3:**
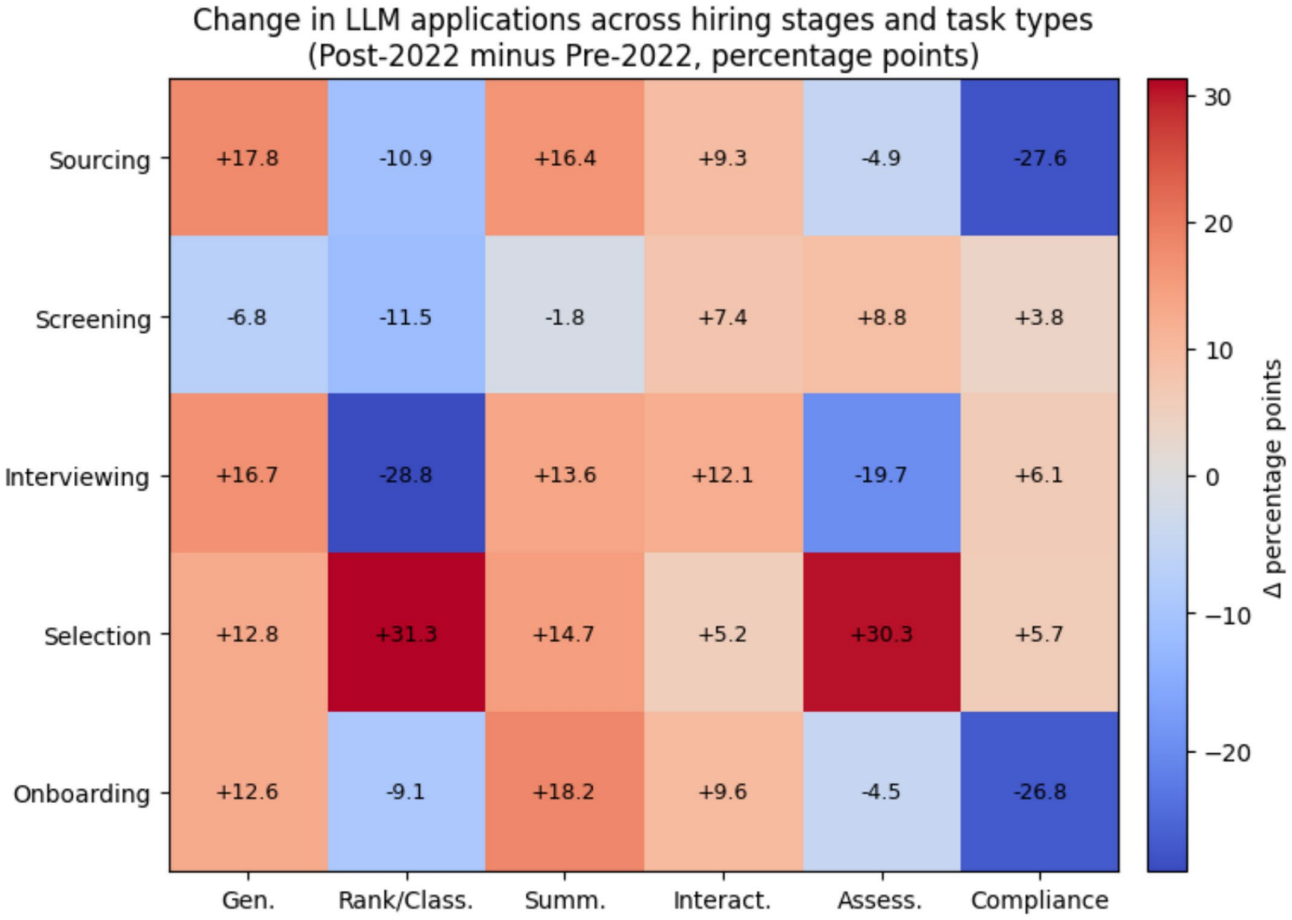
Change in LLM applications across hiring stages and task types.

Screening-stage applications focus on résumé parsing, ranking, and summarization. These practices frame candidates as structured data objects. Interview-stage applications introduce LLMs into more interactive and interpretive roles. These include question generation and conversational agents. This shift has important theoretical implications, as illustrated in Figure 3. Earlier algorithmic hiring tools mainly functioned as computational decision aids. By contrast, LLMs act as language-producing systems that shape how candidates are represented, evaluated, and discussed. Based on sense making theory, these systems participate in constructing the narratives through which hiring decisions are justified (Weick, 1995).

**Figure 4:**
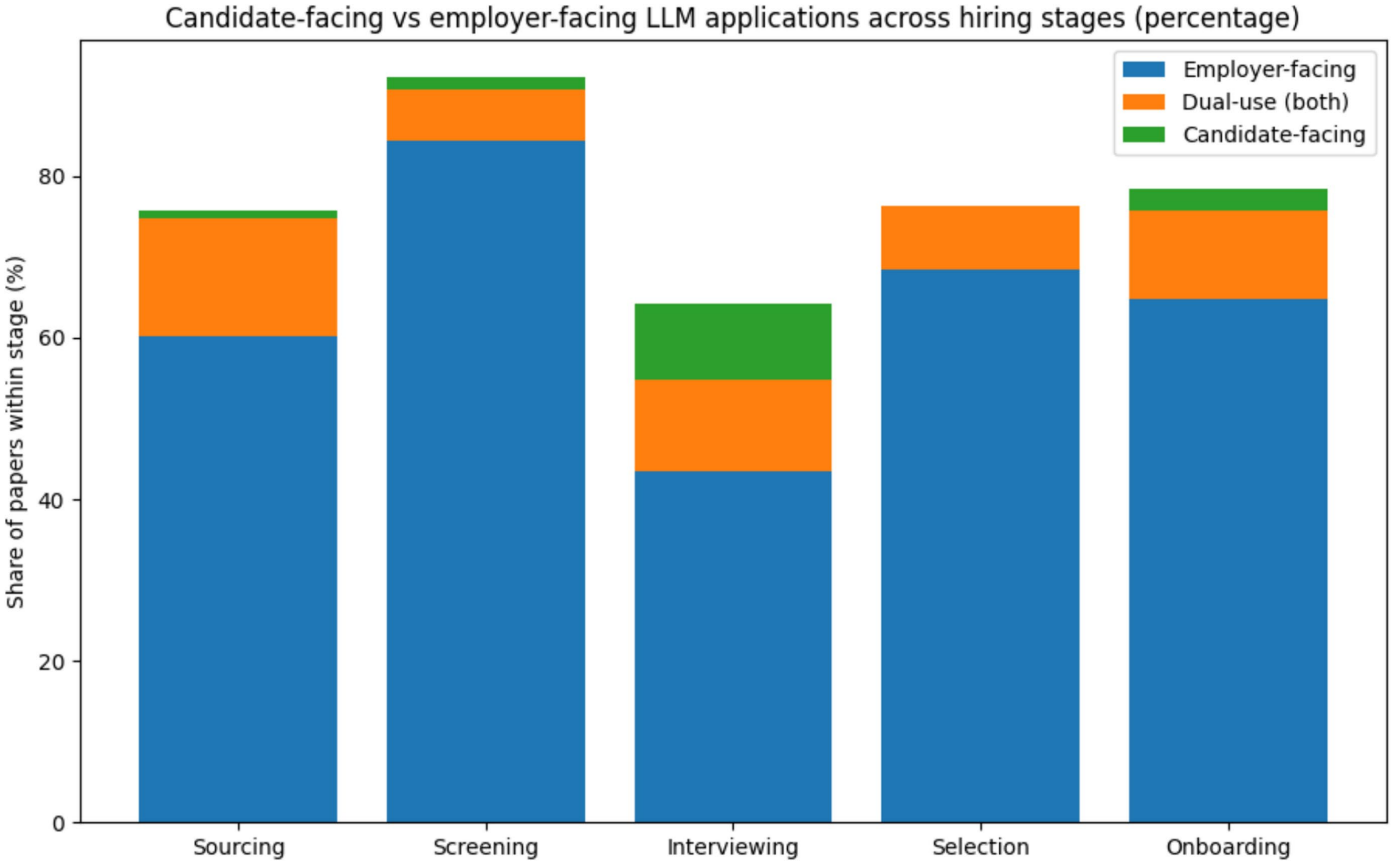
Candidate-facing vs. employer-facing LLM applications.

However, research attention to later stages such as selection and onboarding remains limited. This absence suggests an organizational preference to deploy LLMs where decision responsibility can remain diffuse and reversible. The Figure 4 indicates that approximately 70–75% of reported LLM applications in hiring are employer-facing, while 25–30% are candidate-facing. Employer-facing uses mainly involve résumé screening, candidate ranking, summarization, and drafting of evaluation notes. Candidate-facing uses are concentrated in interview chatbots and automated question answering. This pattern suggests that LLMs are currently adopted primarily to support internal decision efficiency rather than candidate experience. Such back-end–first adoption is consistent with socio-technical theory, which predicts that organizations introduce new technologies first in controllable internal processes (Trist and Bamforth, 1951; Orlikowski and Iacono, 2001). The limited visibility of LLMs to candidates also raises concerns for perceived fairness and transparency, which are central to applicant reactions (Gilliland, 1993; Colquitt et al., 2001).

**Figure 5:**
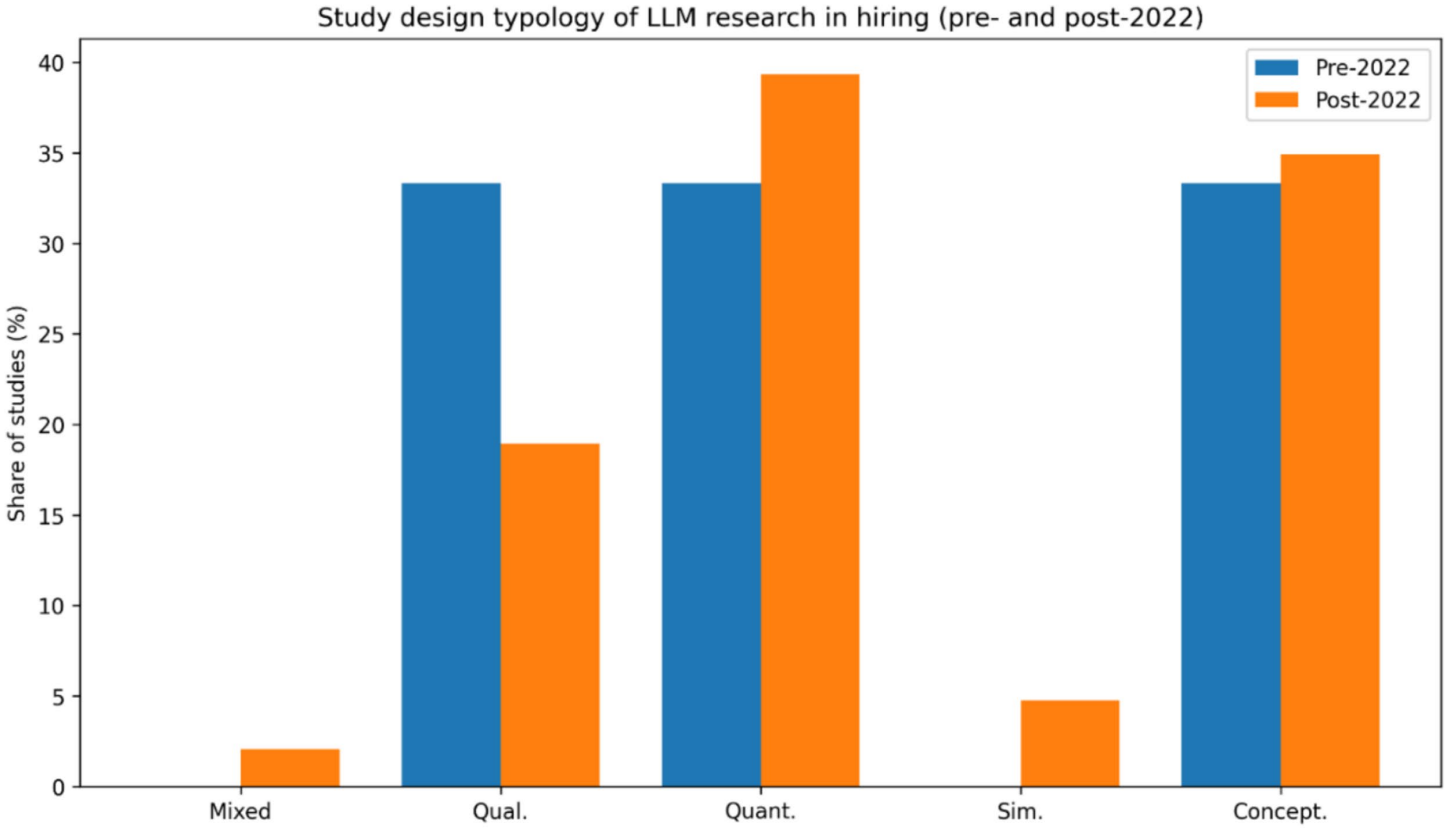
Study design typology of LLM research in hiring [Pre and Post AI diffusion (2022)].

The Figure 5 shows that the literature is dominated by conceptual and quantitative studies, which together account for approximately 65–70% of publications. Experimental or simulation-based designs represent about 20–25%, while field-based and mixed-methods studies remain limited at roughly 10–15%. This distribution indicates that research on LLMs in hiring is still largely exploratory and design-oriented, with relatively little evidence drawn from real organizational deployments. Such patterns are typical of early-stage technological fields, where system development and proof-of-concept evaluations precede large-scale field validation (Orlikowski, 2007). The limited presence of qualitative and mixed-methods work also constrains understanding of how LLMs interact with human judgment and organizational context, which are central to hiring as a socio-technical process (Trist and Bamforth, 1951; Dipboye, 2018). The dominance of conceptual and experimental research indicates that the field remains in an early stage of empirical institutionalization, where technological capabilities are advancing faster than organizational adoption research. This pattern is consistent with information systems research trajectories in which theoretical framing and system development precede field-based validation (Orlikowski and Iacono, 2001). Future studies should prioritize longitudinal and field-based research examining how LLM-supported hiring systems operate in real organizational contexts, including their effects on decision accountability, interpretive practices, and institutional legitimacy.

**Figure 6:**
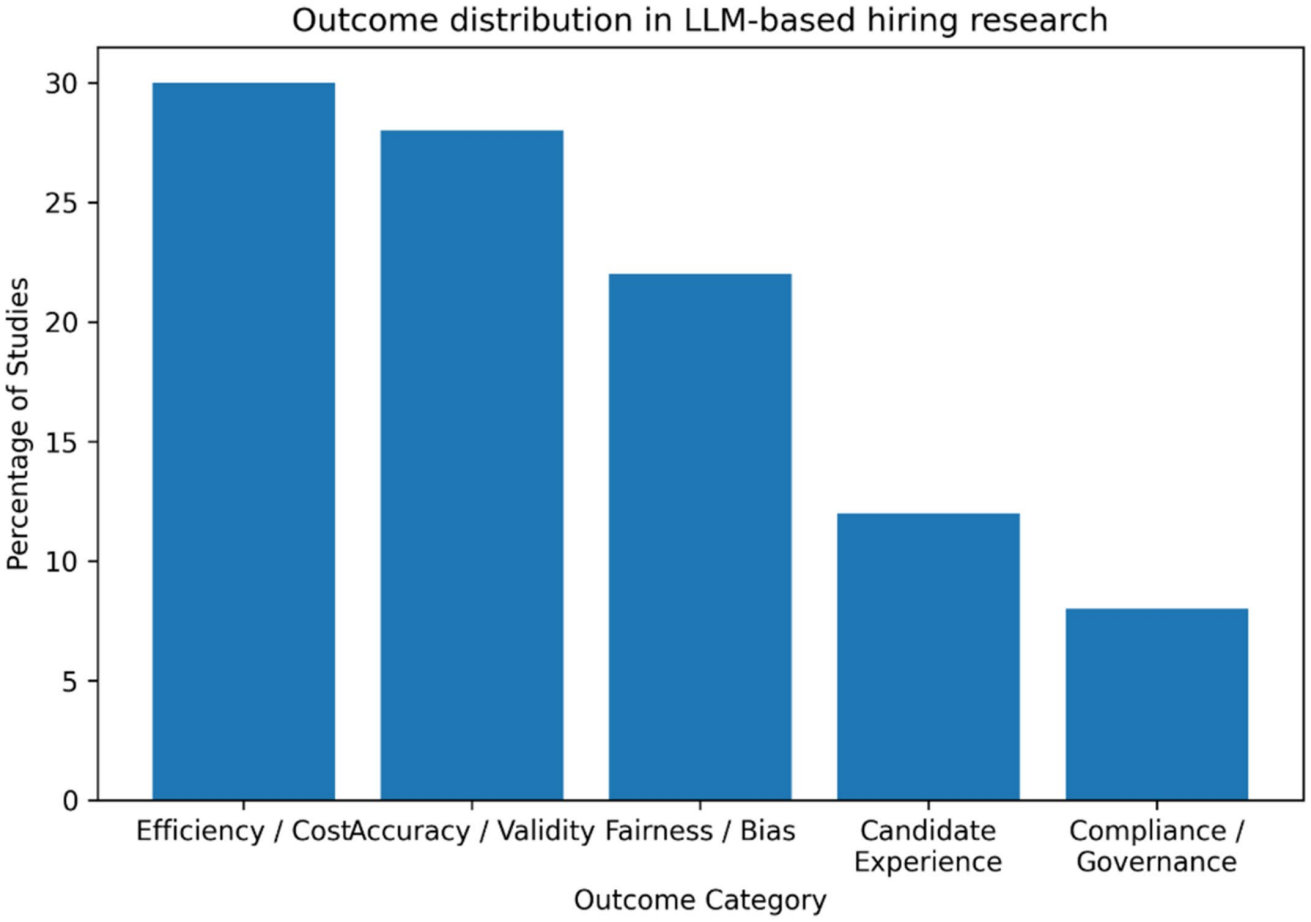
Outcome distribution.

The Figure 6 indicates that outcome assessment in LLM-based hiring research is concentrated on efficiency and performance, which together account for approximately 55–60% of reported outcomes. Fairness and bias-related outcomes represent about 20–25%, while candidate experience and compliance or governance outcomes remain limited at roughly 15–20% combined. This pattern reflects an instrumental orientation in which technologies are primarily evaluated in terms of speed, cost reduction, and predictive quality (March and Simon, 1958; Orlikowski and Iacono, 2001). In contrast, outcomes central to organizational justice and applicant reactions receive comparatively less attention, despite strong evidence that perceived fairness and transparency shape acceptance of selection systems (Gilliland, 1993; Colquitt et al., 2001).

**Figure 7:**
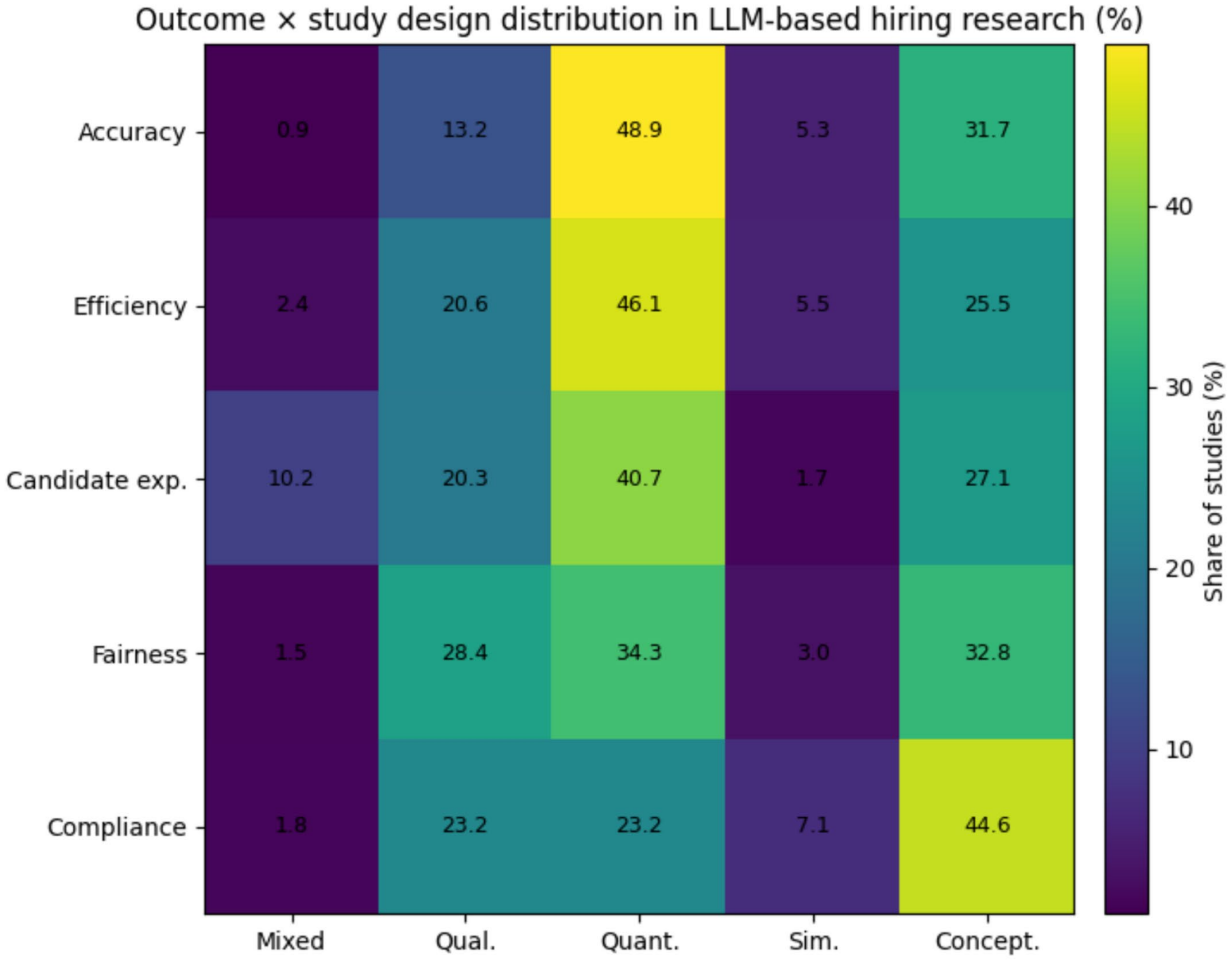
Outcome vs. study design matrix.

The Figure 7 indicates that in conceptual and experimental studies, approximately 60–65% of reported outcomes relate to efficiency and accuracy, while only 15–20% address fairness or governance. In contrast, qualitative and mixed-methods studies devote roughly 45–50% of their outcome focus to fairness, candidate experience, and governance, with the remainder addressing performance-related outcomes. Candidate experience outcomes appear almost exclusively in qualitative and mixed-methods work and represent <10% of outcomes in quantitative or simulation-based studies. This pattern suggests that outcome selection is strongly shaped by methodological choice, with technical designs favoring measurable performance metrics and socially embedded outcomes requiring methods that capture perception and context (March and Simon, 1958; Gilliland, 1993; Trist and Bamforth, 1951). This emphasis on efficiency and performance reflects a technical orientation that may overlook the institutional and perceptual dimensions of hiring decisions. Organizational theory suggests that decision legitimacy and fairness perceptions play a critical role in shaping acceptance and trust in selection systems (Gilliland, 1993; Colquitt et al., 2001; Suchman, 1995). Future research should integrate technical performance evaluation with organizational and behavioral outcomes, including candidate perceptions, decision transparency, and the institutional consequences of LLM-mediated hiring practices.

**Figure 8:**
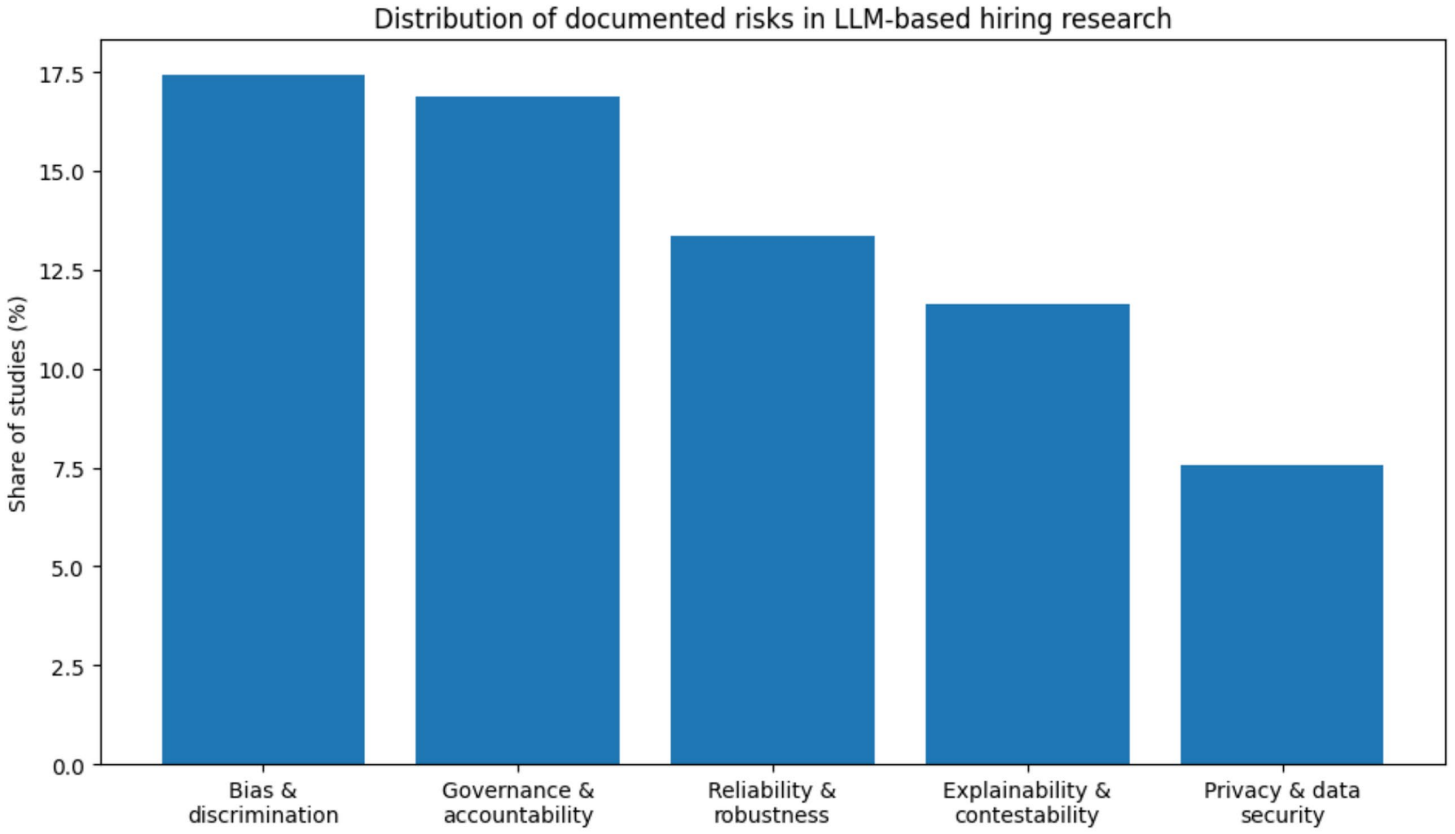
Distribution of documented risk.

The Figure 8 shows that bias and discrimination risks are the most frequently discussed, accounting for approximately 35–40% of all reported risk mentions. Explainability and transparency risks represent about 20–25%, followed by privacy and security risks at roughly 15–20%. Reliability-related risks (e.g., hallucination, inconsistency) and accountability risks together account for approximately 15–20%. This distribution indicates that the literature is strongly oriented toward fairness-related concerns, while issues of responsibility and system dependability receive comparatively less attention. Such patterns mirror broader debates in algorithmic decision-making, where ethical risk identification often precedes systematic evaluation of governance and accountability mechanisms (Barocas and Selbst, 2016; Kroll et al., 2017; Raji et al., 2020).

**Figure 9:**
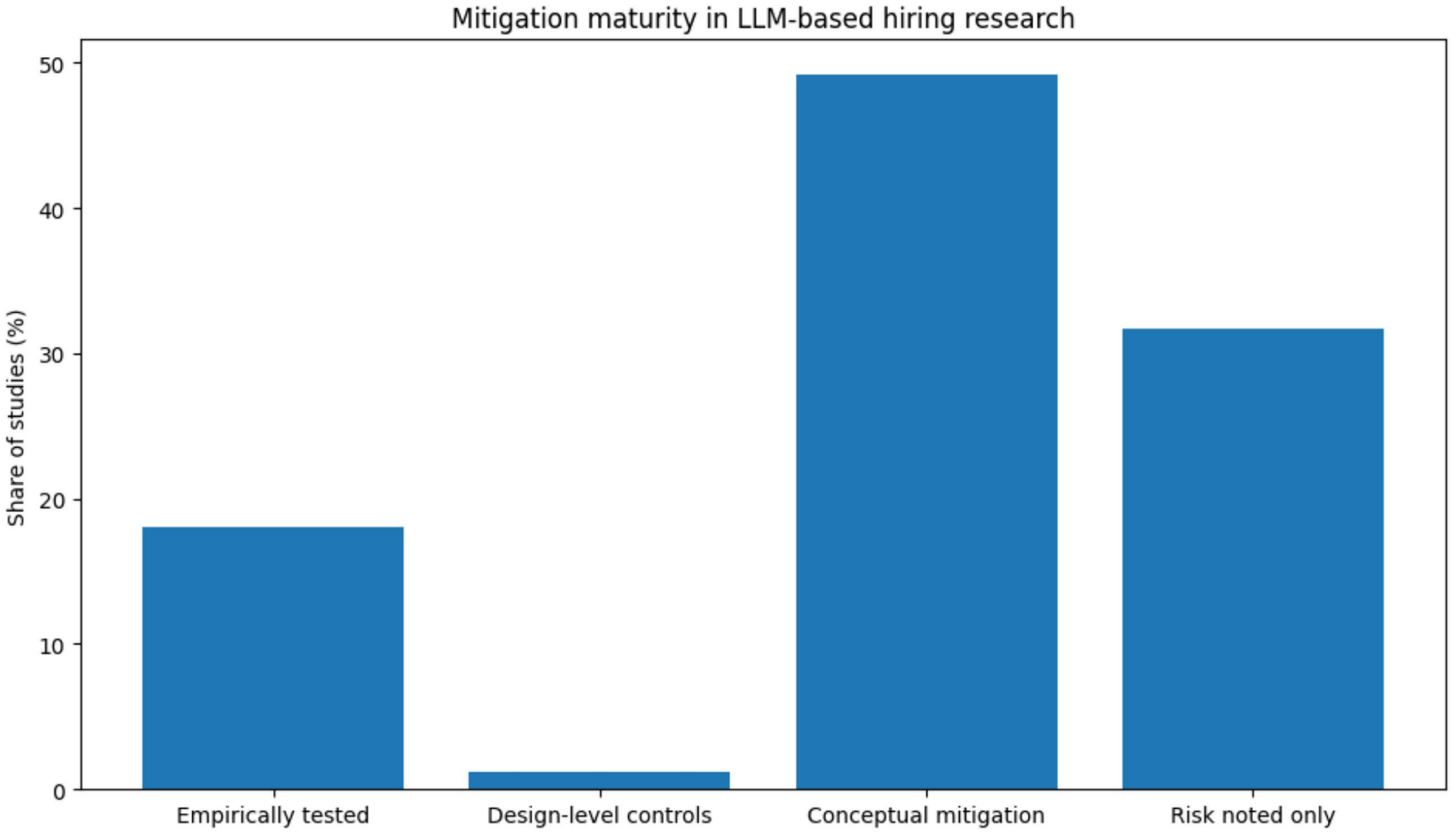
The mitigation maturity in LLM based hiring.

These five empirically identified risk categories align closely with established governance dimensions defined in the NIST AI Risk Management Framework (NIST, 2023), supporting comparability with broader AI governance literature (Table 1). The Figure 9 indicates that most mitigation strategies discussed in the literature remain at an early or conceptual stage, with approximately 60–65% of mitigation mentions describing proposed rather than empirically tested controls. Human-in-the-loop oversight and bias auditing together account for roughly 40–45% of all reported mitigation approaches, while documentation and transparency mechanisms represent about 25–30%. Governance frameworks and organizational policies appear least frequently, at approximately 15–20%. This pattern suggests that while risk awareness is high, mitigation practices are not yet institutionalized or systematically evaluated. Similar gaps between ethical principles and operational governance have been documented in broader algorithmic accountability research (Raji et al., 2020; Veale and Borgesius, 2021).

**Table 1:** Alignment between lexicon-derived risk categories and governance dimensions defined in the NIST AI Risk Management Framework (NIST, 2023).

Risk category identified in this review	Description in hiring context	Corresponding NIST AI RMF risk dimension
Bias and discrimination	Systematic differences in candidate evaluation due to model behavior or training data	Fairness – Harmful bias and discrimination
Explainability and transparency	Limited ability to interpret, understand, or justify model-supported hiring outputs	Transparency and explainability
Privacy and security	Risks related to exposure, misuse, or protection of candidate personal data	Privacy and data security
Reliability	Inconsistent, incorrect, or hallucinated outputs affecting decision quality	Validity and reliability
Accountability	Lack of clear responsibility, oversight, or contestability of LLM-supported hiring decisions	Accountability and governance

**Figure 10:**
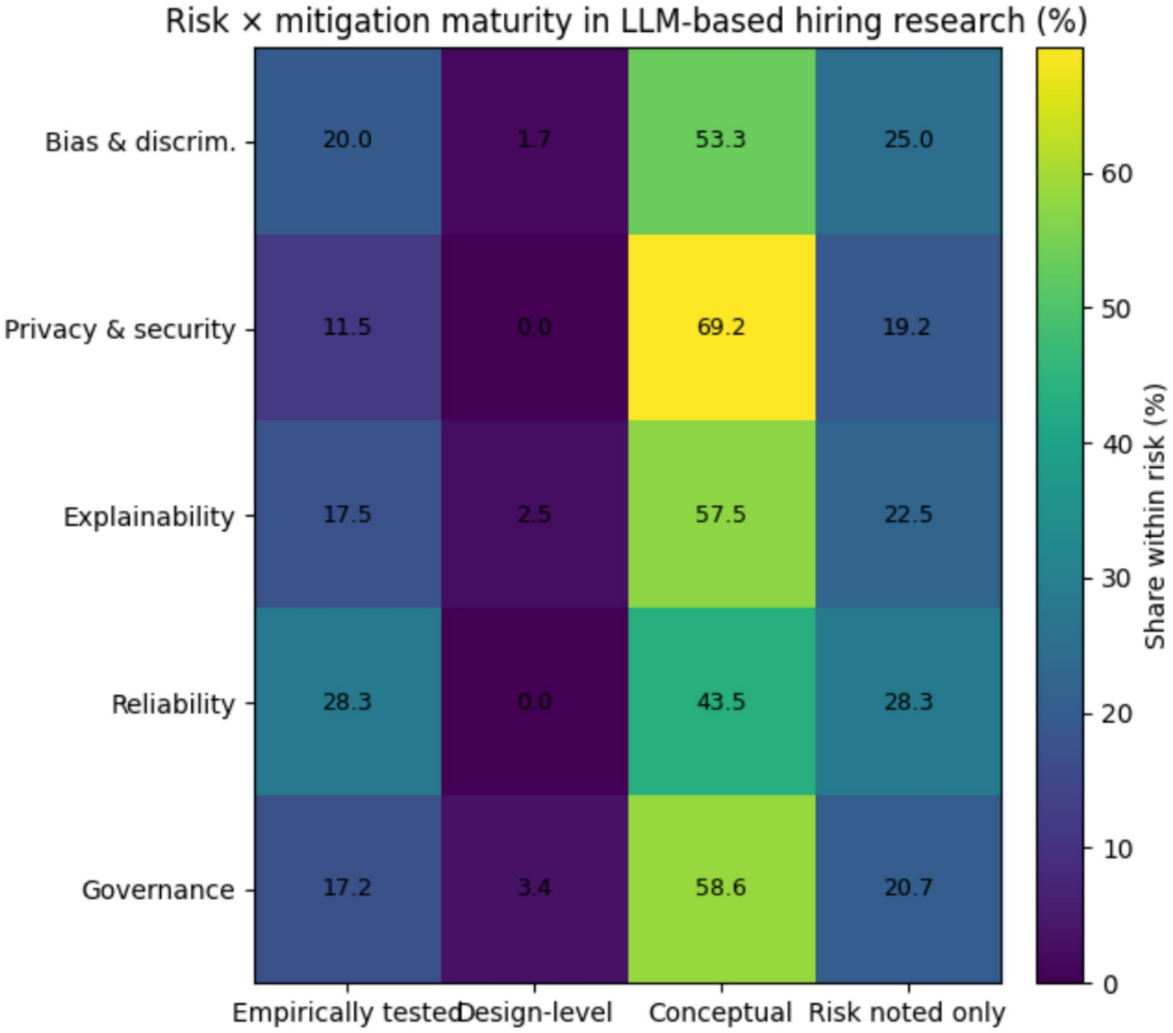
Mitigation maturity vs. risk in LLM based hiring.

Figure 10 shows an uneven alignment between risk categories and mitigation maturity. For bias and discrimination risks, approximately 50–55% of studies propose mitigation strategies, but only 15–20% report empirical testing of these controls. For privacy and security risks, around 40% of studies mention mitigations, with fewer than 15% providing evidence of implementation. Explainability risks show a similar pattern, with about 45% proposing transparency mechanisms and roughly 15% evaluating them empirically. Accountability risks display the weakest alignment, with <25% of studies proposing concrete governance mechanisms. This pattern indicates that mitigation efforts largely remain conceptual rather than operational. The gap between risk identification and empirically validated mitigation reflects broader challenges in governing algorithmic decision systems, where accountability mechanisms often lag technological capability (Raji et al., 2020; Veale and Borgesius, 2021). From an institutional perspective, the legitimacy of LLM-supported hiring decisions depends not only on system performance but also on demonstrable governance and oversight mechanisms (Scott, 2014; Suchman, 1995). Future research should empirically evaluate governance practices such as auditing, human oversight, and documentation to determine how they influence decision accountability and organizational trust. Consistent with algorithmic governance research, risk identification currently outpaces evidence on whether safeguards meaningfully change decision processes (Kroll et al., 2017; Raji et al., 2020; Veale and Borgesius, 2021). The governance and accountability risks identified in this review align closely with emerging regulatory frameworks governing AI use in employment. In particular, the European Union Artificial Intelligence Act classifies AI systems used in hiring and employment decisions as high-risk applications subject to requirements for risk management, documentation, transparency, and human oversight (Veale and Borgesius, 2021). These requirements correspond directly to the risks identified in this review, including transparency limitations, accountability gaps, and insufficiently validated mitigation mechanisms. This alignment indicates that the governance challenges discussed in the academic literature are also reflected in emerging regulatory priorities, highlighting the importance of empirically grounded research on governance practices for LLM-assisted hiring systems. Similarly, employment regulators such as the U. S. Equal Employment Opportunity Commission have emphasized that automated hiring tools must comply with existing anti-discrimination and accountability requirements, reinforcing the importance of transparency, fairness, and human oversight in AI-supported employment decisions (Equal Employment Opportunity Commission, 2023).

**Figure 11:**
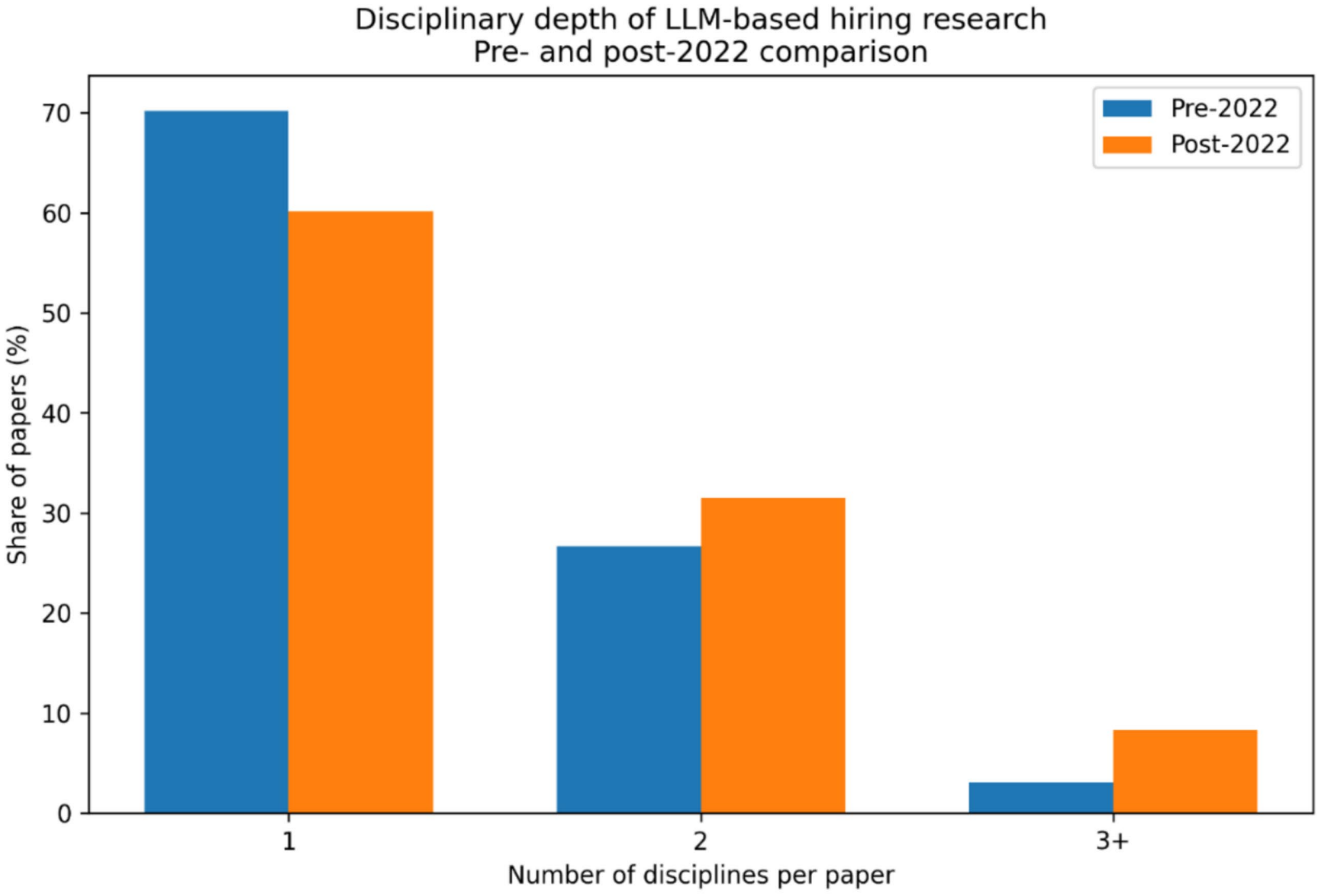
The disciplinary depth per paper.

The Figure 11 indicates that approximately 65–70% of studies are single-disciplinary, while 20–25% draw on two disciplines, and only 5–10% involve three or more disciplines. This distribution suggests that most research on LLMs in hiring is still conducted within disciplinary silos, with limited theoretical and methodological integration. Such patterns are typical of emerging technological fields, where early work is anchored in dominant home disciplines before deeper interdisciplinarity develops (Abbott, 2001). The limited share of multi-disciplinary studies constrains the development of integrative frameworks that connect technical performance, human judgment, organizational context, and governance. These findings underscore the need for more genuinely interdisciplinary research to address hiring as a socio-technical system (Trist and Bamforth, 1951; Orlikowski, 2007).

**Figure 12:**
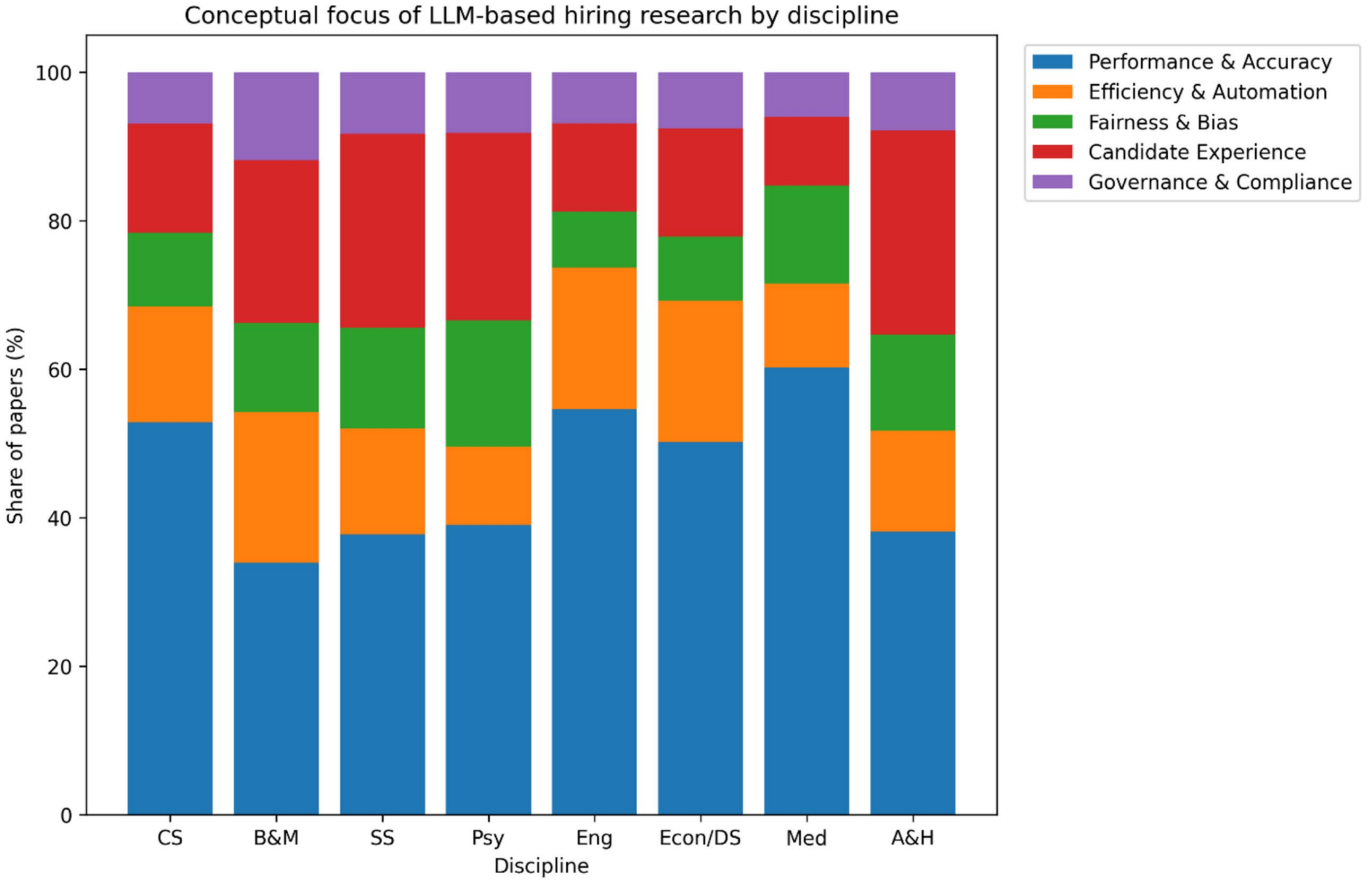
Conceptual focus of LLM-based hiring research by discipline.

The Figure 12 shows clear differences in conceptual emphasis across disciplines. Studies in computer science and engineering focus primarily on performance and efficiency outcomes, which account for approximately 60–65% of their conceptual framing. In contrast, research in the social sciences and psychology places greater emphasis on fairness, bias, and candidate experience, representing about 55–60% of conceptual focus. Business and management studies more frequently emphasize governance, compliance, and organizational adoption, accounting for roughly 45–50% of their conceptual framing. These patterns indicate that disciplinary traditions strongly shape what aspects of LLM-based hiring are foregrounded. While this diversity enriches the field, it also contributes to fragmented knowledge development, as different disciplines prioritize different problem definitions and success criteria (Abbott, 2001; Orlikowski and Iacono, 2001). Greater theoretical integration across disciplines is therefore needed to develop more holistic models of LLM use in hiring.

**Figure 13:**
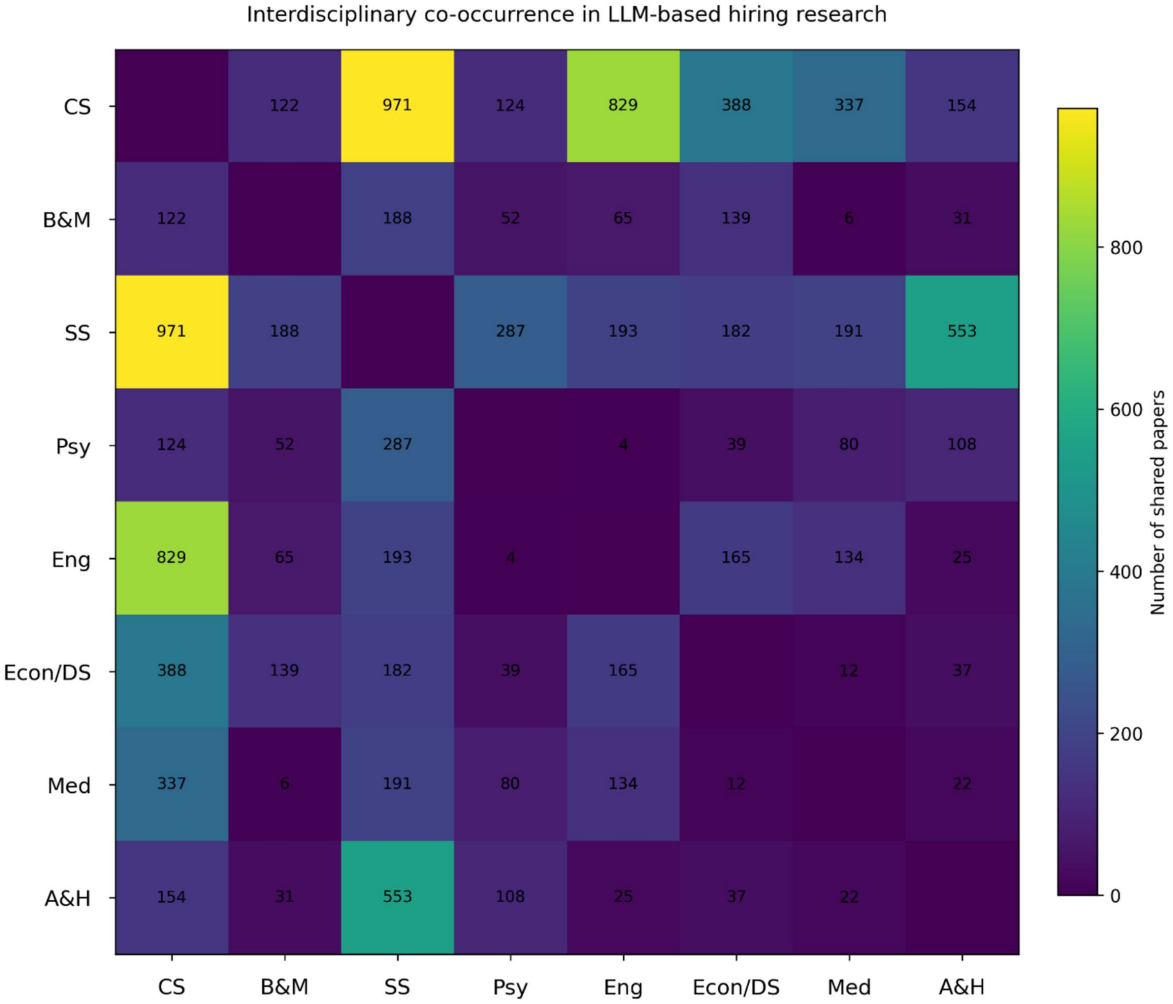
Interdisciplinary co-occurrence in LLM based hiring.

Figure 13 shows that the strongest disciplinary co-occurrence occurs between computer science and engineering, followed by links between computer science and social sciences. Co-occurrence involving psychology, medicine, and arts and humanities is comparatively limited. This pattern indicates that most interdisciplinary collaboration in LLM-based hiring research centers on technical development paired with general social analysis, while behavioral, clinical, and normative perspectives remain weakly integrated. Such boundary-maintaining collaboration is common in emerging technological fields, where disciplines interact but retain distinct problem framings and methods (Abbott, 2001). This limited interdisciplinary integration suggests that the theoretical development of LLM-based hiring research remains structurally fragmented. Technical research emphasizes system performance, while organizational and governance research focuses on institutional implications, resulting in parallel rather than cumulative knowledge development (Abbott, 2001; Orlikowski and Iacono, 2001). Future research should develop integrative theoretical frameworks that connect computational capability, human judgment, and organizational governance to better explain how LLMs reshape hiring as a socio-technical decision system.

Across all four research questions, a consistent pattern emerges. LLMs are being introduced into hiring as technical solutions to organizational problems, while their deeper socio-technical implications remain insufficiently integrated into empirical research. The literature reflects what sociologists of technology describe as a technology-first trajectory, in which technical capabilities outpace governance arrangements and theory lags behind practice (Orlikowski, 2007; Winner, 2017). By reframing hiring as a socio-technical decision system rather than as a purely computational task, this review highlights the need for research that bridges system design, human judgment, organizational context, and regulatory accountability. Taken together, the findings suggest that existing theories of automation, decision-making, and governance require extension to account for language-based AI systems. The reviewed literature suggests that LLMs may influence how evaluative narratives are constructed and interpreted, with potential implications for interpretive authority and accountability relationships. Accordingly, this review supports a shift from viewing AI in hiring as a decision aid toward conceptualizing it as a discursive actor within organizational decision systems. Future theoretical work should integrate socio-technical systems theory, sense making, organizational justice, and algorithmic governance into unified frameworks capable of explaining how LLMs influence hiring outcomes in practice.

## 4 Theoretical implications

As a scoping review, this study maps patterns in existing literature rather than testing causal effects or empirically validating organizational outcomes. The reviewed studies provide empirical evidence regarding where LLMs are applied, what outcomes are measured, and what risks and mitigation strategies are discussed. However, many studies remain conceptual, simulation-based, or design-oriented, with limited field-based validation. Accordingly, interpretations of LLMs as socio-technical decision intermediaries or language-mediated actors should be understood as theory-informed inferences derived from observed research patterns rather than as empirically established organizational effects. This distinction ensures that the review separates descriptive synthesis from theoretical interpretation, consistent with scoping review methodology (Tricco et al., 2018).

This review tries to advance theoretical understanding of large language models (LLMs) in hiring by clarifying their emerging role as socio-technical decision intermediaries embedded within organizational evaluation systems. The concentration of LLM use in early hiring stages suggests incremental integration that redistributes cognitive and interpretive work while preserving human evaluative authority. This pattern aligns with socio-technical systems theory, which emphasizes that technological artifacts reshape decision processes through interaction with organizational structures rather than replacing human judgment completely (Trist and Bamforth, 1951; Baxter and Sommerville, 2011; Orlikowski, 2007). At the same time, the literature reveals a systematic gap between technological development and theoretical engagement with hiring as an institutional and socially integrated process. Many studies emphasize performance and efficiency but do not engage with established theories concerning fairness, legitimacy, and organizational decision-making (Gilliland, 1993; Colquitt et al., 2001; March and Simon, 1958). This exclusion limits theoretical understanding of how language-mediated AI systems interact with institutional norms and accountability structures that govern hiring decisions (Scott, 2014; Suchman, 1995). The review also highlights structural fragmentation across disciplinary domains. Computer science research primarily conceptualizes LLMs as computational tools, whereas organizational and governance research emphasizes socio-institutional implications, including legitimacy, accountability, and fairness risks (Abbott, 2001; Orlikowski and Iacono, 2001; Raji et al., 2020). This fragmentation constrains cumulative theory development and reflects the interdisciplinary but structurally disconnected nature of emerging technological research domains. Finally, theoretical claims regarding the transformative organizational impact of LLMs currently exceed the empirical evidence base, which remains largely conceptual or simulation-driven. This pattern is consistent with early-stage technological fields, where conceptual framing precedes empirical institutionalization and organizational embedding (Weick, 1995; Parasuraman and Riley, 1997). Collectively, these findings indicate that LLMs are best understood not simply as automation tools but as socio-technical artifacts embedded within institutional decision systems, whose implications depend on their interaction with organizational structures, governance mechanisms, and human evaluation practices. By identifying these structural theoretical gaps, disciplinary silos, and conceptual inconsistencies, this review contributes to theory development by clarifying the conceptual positioning of LLMs in hiring and establishing priorities for empirically grounded interdisciplinary research.

## 5 Conclusion

This scoping review mapped how large language models (LLMs) are conceptualized, studied, and evaluated in hiring, including their placement across the hiring pipeline, the evidence used to assess them, the risks and governance mechanisms discussed, and the disciplinary structures shaping the field. The findings reveal a rapidly expanding literature that remains uneven in empirical maturity and theoretical integration. The reviewed studies suggest that LLMs function not only as computational tools but as language-mediated decision-support systems that shape how candidate information is organized, interpreted, and justified. However, the literature remains heavily oriented toward efficiency and performance outcomes, while organizational, institutional, and governance implications receive comparatively less empirical attention. Although risks related to bias, transparency, and accountability are widely recognized, mitigation strategies are largely conceptual and rarely evaluated in real organizational settings. Research on LLM-based hiring is increasingly interdisciplinary, yet integration across disciplinary perspectives remains limited. Advancing the field will require theory-informed, field-based research that connects technical capability with organizational context, human judgment, and governance mechanisms. This review provides a structured foundation for understanding the current state of the literature and clarifies priorities for future interdisciplinary research on responsible LLM use in hiring.

## 6 Implications for theory and practice

This scoping review has implications for theory, organizational practice, and governance. From a theoretical perspective, the findings indicate that large language models function not only as computational tools but as socio-technical artifacts that shape how candidate information is interpreted and justified within organizational decision systems. This extends existing socio-technical and organizational decision-making frameworks by highlighting the role of language-mediated AI in interpretive and evaluative processes (Orlikowski and Iacono, 2001; Weick, 1995; Scott, 2014).

From an organizational perspective, the concentration of LLM use in efficiency-oriented tasks, alongside limited empirical evaluation of governance mechanisms, suggests that responsible adoption requires clear oversight, transparency, and accountability structures. Governance practices such as human review and auditing may be important for maintaining decision legitimacy and defensibility (Raji et al., 2020; Suchman, 1995).

From a policy perspective, the gap between risk identification and empirically validated mitigation highlights the need for governance frameworks supported by empirical evidence. Research that examines how accountability and oversight mechanisms function in practice can inform both organizational policy and regulatory development for LLM-assisted hiring systems.

## 7 Limitations

This review has several limitations. First, it is based on Scopus-indexed literature, which may underrepresent relevant studies published in non-indexed journals, practitioner outlets, preprints, and policy reports. As a result, some industry-led and proprietary evaluations of LLM-based hiring tools may not be captured. Second, coding relied on titles, abstracts, and keywords using a lexicon-based, rule-driven approach. While this supports transparency and reproducibility, it may miss nuanced discussions or implicit theoretical positions that appear only in full texts. Studies using alternative terminology may also be undercounted. Third, disciplinary classification was derived from search strategies and metadata and may not perfectly reflect the intellectual orientation of individual papers. Interdisciplinary work may therefore be unevenly represented. Fourth, the fast-evolving nature of generative AI introduces temporal limitations. The review reflects the literature up to early 2026, and some findings may change as models, regulations, and organizational practices develop. Finally, as a scoping review, this study does not assess causal effects, comparative effectiveness, or real-world impact. The findings should be interpreted as patterns of research attention rather than as evaluations of system performance or harm.

## 8 Future research directions

The findings of this scoping review highlight the need for research that moves beyond technical system evaluation toward understanding LLM use as an organizational and socio-technical phenomenon. A key priority is the development of empirically grounded research examining how LLM-assisted hiring systems are implemented, governed, and integrated within real organizational decision processes. Such work would strengthen the empirical foundation of the field and clarify how LLM-supported decision-making interacts with institutional norms, accountability structures, and organizational practices. Future research should also focus on developing integrative theoretical frameworks that connect computational capability with human judgment, organizational context, and governance mechanisms. Current research remains fragmented across disciplinary perspectives, limiting cumulative theory development. Integrative approaches that bridge technical, organizational, and governance perspectives are needed to explain how language-mediated AI systems influence decision interpretation, accountability, and legitimacy in hiring. Finally, as LLM capabilities and regulatory environments continue to evolve, research should examine how organizational adoption, governance practices, and institutional expectations co-evolve over time. Such work will be essential for understanding the long-term implications of LLM integration and for supporting the responsible and effective use of language-based AI in employment decision systems.
