# Supplementary material for: Mapping the use of large language models in hiring decisions: a scoping review
Source: Front Artif Intell. 2026 Mar 13;9:1798519. doi: 10.3389/frai.2026.1798519 (PMC13021839; doi:10.3389/frai.2026.1798519)
Supplement: Supplementary file 1 [file Data_Sheet_1.PDF]

<u>Supplementary File :</u>

Figure No.	Figure Title (as in manuscript)	Lexicon Dimensions Used
Figure 1	Growth of LLM in Hiring research (2020–2025)	Bibliographic metadata only (publication year; funding statement)
Figure 2	Hiring stage at which LLM is used	Hiring Stage (Table A1) + Task Type (Table A2)
Figure 3	Change in LLM applications across hiring stages and task types	Hiring Stage (Table A1) + Task Type (Table A2)
Figure 4	Candidate-facing vs employer-facing LLM applications	Application Orientation (Table A3)
Figure 5	Study design typology of LLM research in hiring (pre and post AI diffusion 2022)	Study Design (Table A4)
Figure 6	Outcome distribution	Outcomes (Table A5)
Figure 7	Outcome vs study design matrix	Outcomes (Table A5) + Study Design (Table A4)
Figure 8	Distribution of documented risk	Risk Categories (Table A6)
Figure 9	Mitigation maturity in LLM-based hiring	Mitigation Strategies (Table A7)
Figure 10	Mitigation maturity vs risk in LLM-based hiring	Risk Categories (Table A6) + Mitigation Strategies (Table A7)
Figure 11	Disciplinary depth per paper	Discipline Assignment (Table A8)
Figure 12	Conceptual focus of LLM-based hiring research by discipline	Conceptual Focus (Table A9) + Discipline Assignment (Table A8)
Figure 13	Interdisciplinary co-occurrence in LLM-based hiring	Discipline Assignment (Table A8)

Table A1. Lexicon for Hiring Stage (Figure: LLM applications across hiring stages)

**Hiring Stage**	**Lexicon Terms (case-insensitive OR match)**
Sourcing	"job ad", "job advertisement", "vacancy description", "sourcing", "talent sourcing", "outreach", "employer branding"
Screening	"resume screening", "cv screening", "applicant screening", "shortlisting", "candidate ranking", "application filtering"
Interviewing	"interview", "interview questions", "virtual interview", "interview chatbot", "conversational interview", "automated interview"
Selection	"hiring decision", "final selection", "candidate evaluation", "decision support", "hiring recommendation"
Onboarding	"onboarding", "new hire", "employee integration", "orientation", "post-hire"

Coding rule: Binary presence per paper.

Table A2. Lexicon for Task Type (Figure: Hiring Stage × Task Type Heatmap)

**Task Type**	**Lexicon Terms**

Generation	"generate", "draft", "create", "write", "compose"
Ranking / Classification	"rank", "score", "classify", "classification", "predict", "label"
Summarization	"summarize", "summary", "summarisation", "extract key", "condense"
Interaction	"chatbot", "conversation", "dialogue", "q&a", "interactive"
Assessment	"assess", "evaluation", "rating", "rubric", "measure"
Compliance Support	"compliance", "legal", "regulation", "audit", "policy"

Table A3. Lexicon for Candidate-Facing vs Employer-Facing (Figure: Candidate vs Employer Facing Applications)

Orientation	Lexicon Terms
Candidate-facing	"chatbot", "candidate interface", "virtual assistant", "q&a", "conversational agent", "candidate support"
Employer-facing	"resume screening", "shortlisting", "ranking", "decision support", "evaluation", "summary for recruiter"

Table A4. Lexicon for Study Design (Figure: Study Design Typology)

Study Design	Lexicon Terms
Conceptual	"conceptual", "framework", "perspective", "theoretical", "model"
Experimental / Simulation	"experiment", "simulation", "benchmark", "laboratory study", "synthetic data"
Field Study	"case study", "real-world", "organizational deployment", "industry study", "field study"
Mixed Methods	"mixed methods", "qualitative and quantitative", "survey and interview"

Dominant-category rule: Empirical overrides conceptually if both appear.

Table A5. Lexicon for Outcomes (Figure: Outcome Distribution)

Outcome Category	Lexicon Terms
Efficiency / Cost	"efficiency", "time saving", "speed", "cost", "productivity"
Accuracy / Validity	"accuracy", "precision", "recall", "performance", "validity"
Fairness / Bias	"fairness", "bias", "discrimination", "equity", "disparate impact"
Candidate Experience	"candidate experience", "applicant reaction", "trust", "satisfaction", "perception"
Compliance / Governance	"compliance", "regulation", "legal", "accountability", "governance"

Table A6. Lexicon for Risks (Figure: Distribution of Risks)

Risk Category	Lexicon Terms
Bias & Discrimination	"bias", "discrimination", "unfair", "inequality"
Privacy & Security	"privacy", "data protection", "security", "leakage"
Explainability	"explainability", "interpretability", "transparency"
Reliability	"hallucination", "error", "robustness", "inconsistency"
Accountability	"responsibility", "liability", "accountability"

Table A7. Lexicon for Mitigation / Controls (Figure: Mitigation Maturity)

Mitigation Type	Lexicon Terms
Human-in-the-loop	"human oversight", "manual review", "human in the loop"
Bias Audit	"bias audit", "fairness audit", "bias testing"

Documentation	"documentation", "model card", "datasheet"
Transparency Tools	"explainable ai", "transparent model"
Governance Framework	"policy", "governance framework", "standards"

Table A8. Lexicon for Discipline Assignment (Figure: Disciplinary Depth & Co-occurrence)

**Discipline**	**Lexicon Terms**
Computer Science	"computer science", "machine learning", "artificial intelligence"
Engineering	"engineering", "systems engineering"
Social Sciences	"social science", "sociology", "social"
Business & Management	"management", "human resource", "organization"
Psychology	"psychology", "behavioral", "cognitive"
Medicine	"medical", "healthcare", "clinical"
Arts & Humanities	"ethics", "philosophy", "humanities"

Table A9. Lexicon for Conceptual Focus (Figure: Conceptual Focus by Discipline)

Concept	Lexicon Terms
Performance	"accuracy", "efficiency", "performance"
Fairness	"fairness", "bias", "equity"
Candidate Experience	"trust", "satisfaction", "perception"
Governance	"compliance", "regulation", "accountability"

Full Search Strategy

Database: Scopus (Elsevier)

Search Querry:

TITLE-ABS-KEY (("large language model*" OR LLM* OR "generative AI" OR "generative artificial intelligence" OR ChatGPT OR GPT* OR "language model*" OR "foundation model*") AND (hiring OR recruit* OR "personnel selection" OR screening OR "talent acquisition" OR "job applicant*" OR "employment decision*" OR interview* OR assessment*)) AND PUBYEAR > 2019 AND PUBYEAR < 2026 AND (LIMIT-TO (DOCTYPE , "ar") OR LIMIT-TO (DOCTYPE , "re") OR LIMIT-TO (DOCTYPE , "ed")).

:

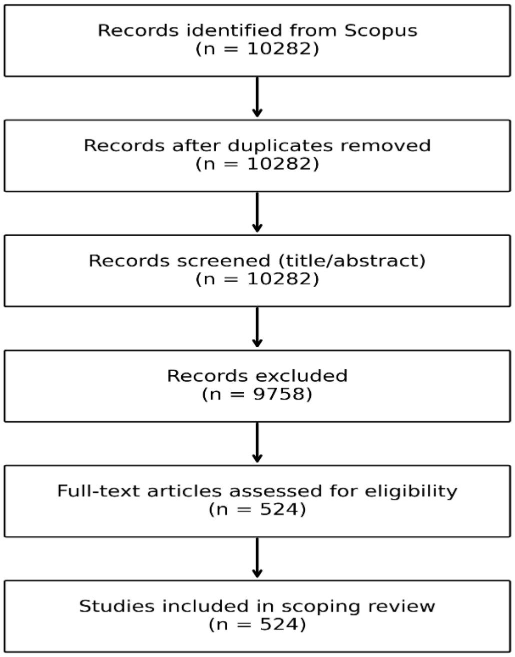

Figure 1. PRISMA-ScR flow diagram of study selection

Table 1: Inclusion exclusion criteria

Inclusion criteria	Exclusion Criteria
1.Explicitly addressed large language models or generative language models<br>2. Examined applications in hiring, recruitment, selection, or related employment decision contexts<br>3.Peer-reviewed journal articles<br>Were published in English	1. Did not involve hiring or personnel selection contexts<br>2. Focused on general AI without explicit reference to large language models<br>3. Preprints, editorials, conference abstracts, dissertations, or technical reports<br>4. written in English
